# Necrotising Myositis, the Deadly Impersonator

**DOI:** 10.1155/2014/485651

**Published:** 2014-11-19

**Authors:** A. Rahman, A. K. Abou-Foul, A. Yusaf, J. Holton, L. Cogswell

**Affiliations:** Department of Plastic and Reconstructive Surgery, Oxford University Hospitals, John Radcliffe Hospital, Headley Way, Oxford OX3 9DU, UK

## Abstract

We report two cases of patients with necrotising myositis who presented initially with limb pain and swelling on a background of respiratory complaints. Patient 1, a previously well 38-year-old female, underwent various investigations in the emergency department for excessive lower limb pain and a skin rash. Patient 2, a 61-year-old female with a background of rheumatoid arthritis and hypertension, presented to accident and emergency feeling generally unwell and was treated for presumed respiratory sepsis. Both deteriorated rapidly and were referred to the plastic surgery team with soft tissue necrosis, impending multiorgan failure and toxaemia. Large areas of necrotic muscle and skin were debrided, which grew group A streptococci, *Streptococcus pyogenes*. Patient 1 had a high above knee amputation of the left leg with extensive debridement of the right. Despite aggressive surgical intervention and microbiological input with intensive care support, patient 2 died. These two cases highlight the importance of early diagnosis and prompt surgical and pharmacological intervention in managing this life-threatening disease. Pain is the primary symptom with skin changes being a late and subtle sign in a septic patient. The Laboratory Risk Indicator for Necrotising Fasciitis (LRINEC) may be of use if there is concern to aid diagnosis of this life-threatening disease.

## 1. Introduction

Group A streptococcus (GAS),* Streptococcus pyogenes*, is a highly virulent pathogen responsible for a wide spectrum of presentations. It can involve a variety of organs including the respiratory tract, skin, heart, and kidneys [1]. Rarely, GAS can cause highly invasive, rapidly progressive, life-threatening infections with extensive destruction of fascia or muscles in the form of necrotising fasciitis (NF) and necrotising myositis, respectively [2]. In this case report we describe two cases of streptococcal toxic shock syndrome (STSS), secondary to fulminant streptococcal necrotising myositis (SNM) with two very different outcomes. We also discuss the clinical picture and management of the disease.

## 2. Case Presentation

Patient 1 was a previously healthy 38-year-old female with an unremarkable past medical history. She was an immunocompetent nonsmoker with a normal body mass index. She presented to her general practitioner (GP) with acute left lower leg pain and swelling. Three weeks prior to this she had a progressive productive cough, swinging fever, and malaise. Her GP suspected a deep venous thrombosis (DVT) and concurrent chest infection and prescribed oral antibiotics. She was referred the same day to a DVT clinic in another institution. No DVT was identified on ultrasound with venous duplex scan but muscle swelling with no sonographically identifiable cause was noted. The DVT team were concerned about the patient and asked for a medical opinion. She was subsequently admitted under the medics.

**Figure 1:**
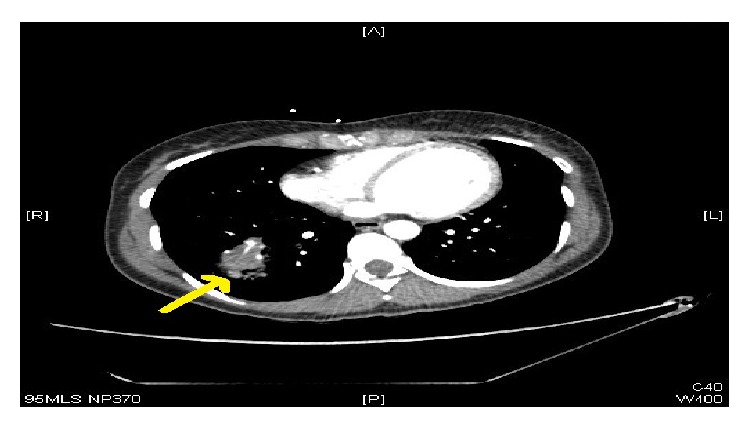
CT scan with contrast showing a right lung abscess, the most likely seeding source for patient 1's invasive muscle infection.

Over the next few hours the patient started to deteriorate and her leg pain and swelling worsened. Only venous duplex was available in the DVT clinic and so the team could not rule out acute limb ischaemia. Once the team had identified diminished distal arterial pulses she was urgently referred and transferred to the vascular surgeons at our regional centre to investigate the possibility of an acute vascular event. However, she arrived haemodynamically unstable. She also complained of mild back pain, so a thromboembolic event associated with an acute aortic dissection was suspected. As dissection was queried, an urgent aortic computed tomography angiogram (CTA) was arranged, which did not identify any evidence of vascular disease. However, a cavity was noted in the lower lobe of her right lung, which was reported as a likely lung abscess (Figure 1).

Patient 2 was a 61-year-old lady with a past history of poorly controlled rheumatoid arthritis and hypertension, but independent in all activities of daily living. She presented to the emergency department with acute pain in both her lower limbs and right arm. Preceding this, she had experienced generalised myalgia, anorexia, vomiting, and flu-like symptoms for 3 days. She had also received an intramuscular steroid injection into her right buttock 5 days earlier for a flare-up of rheumatoid disease. As part of her rheumatoid treatment she also took regular hydroxychloroquine, leflunomide (pyrimidine synthesis inhibitor), and weekly methotrexate alongside the steroid injections for flare-ups and would have been immunosuppressed as a result. On admission she was septic, in fast atrial fibrillation with a heart rate of 150 beats per minute, but maintaining a blood pressure of 130/80 mmHg. Her examination revealed bibasal lung crepitations and a tender, but soft right calf. There was no evidence of pyomyositis at her injection site on her right buttock. She was commenced on digoxin, antibiotics for presumed chest sepsis, and fluid resuscitation. She deteriorated, becoming hypoxic and haemodynamically unstable, and was admitted to the intensive care unit (ICU).

## 3. Investigations

Patient 1 continued to deteriorate clinically with pyrexia, resistant shock, and toxaemia. Arterial blood samples showed acute lactic acidosis with a PH of 7.2 and lactate of 7.9 mmol/L. Her biochemical profile showed acute kidney injury (AKI) with raised creatinine kinase (CK) and acute liver injury as demonstrated in Table 1. The severe pain in her left leg started to subside without systemic improvement. On examination her left leg was found to be completely anaesthetic below the knee and a small blister had developed on top of an area of grey, slightly discoloured skin covering most of the medial aspect of her left lower leg.

**Figure 2:**
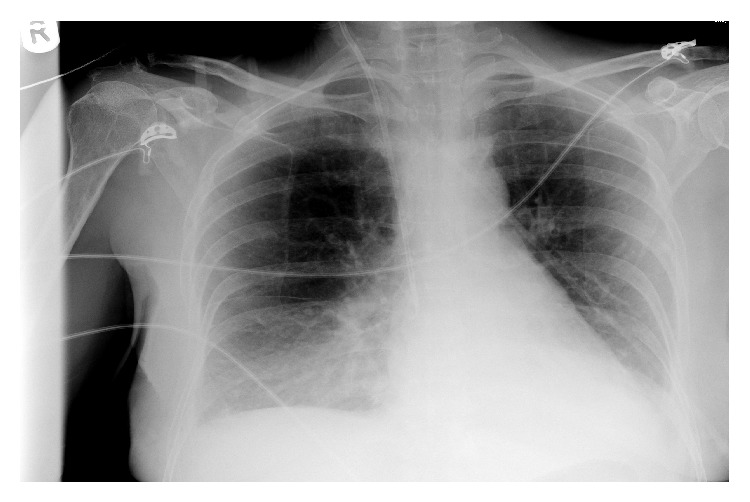
Chest radiograph taken of patient 2 showing right lower lobe consolidation consistent with pneumonia.

Patient 2 also deteriorated, becoming tachycardic, hypotensive, and hypoxic. Bedside echocardiography showed no evidence of ventricular dysfunction and a collapsed inferior vena cave (IVC). A chest radiograph confirmed right lower zone consolidation, which was presumed to be from an infective process and hence diagnosed as pneumonia (Figure 2). Her biochemical profile showed a similar picture to patient 1: hyponatraemia, AKI, raised creatinine kinase, and deranged clotting. Arterial samples similarly showed lactic acidosis with pH of 7.32 and lactate of 8.0.

## 4. Treatment

**Figure 3:**
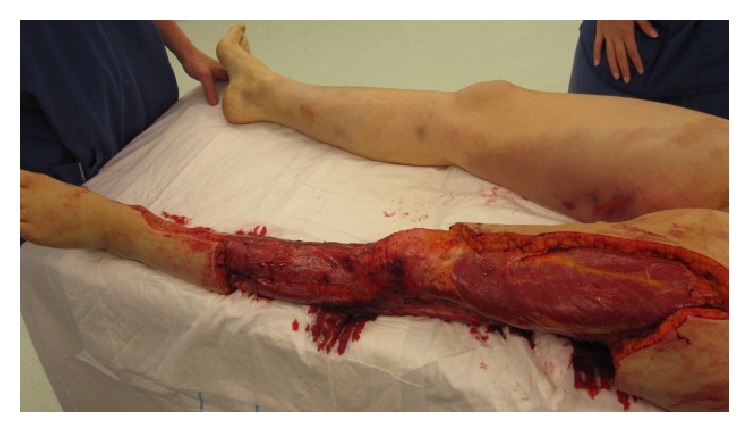
This figure shows patient 1's extensive debridement of the left lower leg following identification of necrotic tissue. This theatre picture at the second look also shows the skin changes developing on the right leg, a late clinical sign.

Patient 1 was urgently referred to the plastic surgical team with suspected necrotising fasciitis. Rhabdomyolysis was considered as a diagnosis given her vastly elevated creatine kinase; however once the skin changes were detected alongside the pyrexia and signs of shock a necrotising condition became the lead diagnosis. Following plastics assessment, including incision of the skin to deep fascia under local anaesthetic and visualisation of necrotic tissue, the patient was resuscitated and started on broad spectrum intravenous antibiotics (meropenem, vancomycin, and clindamycin due to penicillin allergy). She was taken to theatre for emergency surgical exploration and debridement. Almost the entire posterior compartment of the left lower leg was necrotic and hence an extensive excision of the dead tissues was performed (Figure 3). The skin and underlying fascia were far less widely affected and were excised to healthy tissue. Postoperatively, the patient was kept sedated and ventilated on the ICU with multiorgan support. Blood cultures taken before commencing antibiotic therapy demonstrated GAS, as did the many soft tissue samples taken intraoperatively. She continued to deteriorate despite maximum treatment and started to develop new small areas of blistering on her right forearm, right lower leg, and left thigh. She returned to theatre 12 hours later for a second look and further debridement. There was extensive necrosis within the left thigh adductors and gluteus maximus and gluteus medius muscles, which required debridement. The remaining compartments of the left lower leg were all necrotic so an above knee amputation was performed. Skin and soft tissue changes were noted on the right forearm and exploration revealed a small volume of muscle necrosis that needed minimal debridement (approximately 3 cm^2^). Similar skin appearances were observed on her right leg with bruising and mild skin discolouration (Figure 3). Investigative incision through deep fascia yielded necrotic muscle and her right leg and medial thigh needed extensive muscle debridement of the soleus and adductor muscles. Adhesive topical negative pressure dressings were applied to her legs to facilitate wound management. The patient needed eight further visits to theatre in her thirty-eight days of inpatient stay, for dressing changes but minimal further debridement. She remained on the intensive care unit for twenty days for organ support and was transferred to the surgical ward for further observation and treatment.

Patient 2 was treated initially as sepsis, likely secondary to pneumonia in an immunocompromised patient (from her disease modifying antirheumatic drugs and recent steroid injection). She was treated with intravenous co-amoxiclav and clarithromycin and fluid resuscitation and admitted to ICU. After admission her right calf became increasingly swollen, firm, and tender. Erythema on her torso was noted. Her oxygen and inotropic requirements increased and her repeat bloods showed increased CK of 7206 and worsening coagulopathy. She was intubated within 24 hours of being on ICU. A vascular opinion was sought for possible compartment syndrome of her lower limbs. All pulses were present and compartment syndrome was considered unlikely. A computed tomography (CT) of chest, abdomen, and pelvis alongside an ultrasound of the right calf was arranged and antibiotics changed to clindamycin and Tazocin (piperacillin and tazobactam). The CT showed soft tissue swelling in the upper right buttock of unknown significance. Ultrasound of the right lower limb showed oedema and swollen muscles. Blistering around the right groin and right arm was noted at 30 hours after admission. The plastic surgery team reviewed this urgently. Incision of skin to deep fascia under local anaesthetic on ICU showed necrotic soft tissue. Suspecting necrotising fasciitis or myositis, an emergency surgical exploration was arranged. The majority of the medial head of gastrocnemius and half of soleus muscle of her right leg were found to be necrotic. Similar necrotic changes were found within her right biceps muscle with inflamed fascia. Extensive debridement was performed and multiple samples were sent for microbiological analysis. Intraoperative samples yielded GAS (*S. pyogenes*), as with patient 1.

## 5. Outcome and Follow-Up

Patient 1 recovered from her toxaemia and overwhelming sepsis but had large bare areas of exposed muscles and fascia. She was transferred to a nearby burns unit for major skin reconstruction and rehabilitation. She had staged skin allografting with satisfactory results. She is currently awaiting a left leg prosthesis before she undergoes an intensive rehabilitation programme. She would be followed up by cardiothoracic surgeons with a repeat lung CT scan to assess her lung abscess.

Patient 2, in contrast, deteriorated in ICU postoperatively. Her AKI worsened, requiring continuous venovenous hemodiafiltration. Her inotropic requirements increased and metabolic acidosis worsened. Repeat echocardiography showed worsening left ventricular function with an ejection fraction of 10–20%. After discussion with her relatives a decision was made to withdraw inotropes and palliate her. She died just over 48 hours after admission.

## 6. Discussion

Necrotising myositis is a severe but rare form of acute invasive GAS infection with high rates of morbidity and mortality. Overwhelming invasive GAS muscle infections are rare with no more than 30 cases reported over the last century [3]. Most of these cases were reported in the past 40 years, which is consistent with the recent resurgence of highly virulent and pathogenic exotoxin A-producing GAS strains [1, 4].

Streptococcal necrotising myositis (SNM) generally affects healthy, middle-aged patients with the involvement of a single muscle group. This usually represents the thigh, calf, or arm muscle groups [5]. There is usually no history of penetrating trauma, as in our cases, and haematogenous spread by transient bacteraemia from the pharynx to the affected muscle group is the most convincing mechanism of transmission [4, 6]. Blunt trauma has been reported as a risk factor; it increases the binding of GAS to muscle surface proteins, which could act as a localisation factor for SMN [7]. Although our patients did not report any history of trauma, both had symptoms of upper respiratory tract infection prior to hospital admission. This is likely to be the initial source of the GAS bacteraemia.

Diagnosis of SNM is challenging because of its rarity and diverse clinical presentation at early, intermediate, and late stages [3]. Acute SNM is often initially misdiagnosed as acute compartment syndrome, deep venous thrombosis, cellulitis, or even septic arthritis [3]. Characteristically it presents with a prodromal flu-like illness followed by a spontaneous onset of severe pain in the affected muscle group, which is disproportionate to the exhibited clinical signs [5]. Pain is usually accompanied by fever, marked swelling, and nonspecific skin changes, such as erythema, mottling, anaesthesia, and blistering, as the infection extends superficially through the fascial planes and causes coagulation of the perforating blood vessels that supply the overlying skin [4]. As our cases demonstrate, these skin changes appear late in the disease process.

Severe SNM can cause marked systemic toxic effects, namely, the streptococcal toxic shock syndrome (STSS), as defined by the Working Group on Severe Streptococcal Infections criteria [7, 8]. STSS secondary to SNM is a life-threatening host response to GAS superantigens with a mortality rate as high as 80% [9]. It is characterised by refractory shock, fever, rash, skin desquamation, and multi-organ-system dysfunction [1, 8, 10].

Differentiating SNM from NF clinically can be challenging as the skin manifestations are similar [3]. However, in SNM skin involvement will often occur late in the disease process and the changes may be much more subtle than those in NF. Elevated serum CK and magnetic resonance imaging demonstrating muscle inflammation aid the diagnosis of SNM [3, 10]. However, if the condition is suspected, imaging should not delay surgical exploration of the affected muscle group.

The LRINEC is another tool that can be used in the diagnostic conundrum presented by necrotising infections. Focussing on biochemical abnormalities, anaemia, leukophilia, raised CRP, hyponatraemia, raised creatinine, and hyperglycaemia, the LRINEC can aid differentiation of necrotising soft tissue infections from other soft tissue infections (Table 2). A score of greater than 6 points has a negative predictive value of 96% and a positive predictive value of 92% [11]. In hindsight this may have been a useful tool as patient 1 scored 8 and patient 2 scored 11 on admission.

In cases of diagnostic uncertainty, we advocate incision biopsy under local anaesthetic, through fascia for suspected SNM, with samples sent for urgent microscopy and histology [12]. Direct intraoperative observation of the different anatomical planes for necrosis is a powerful early diagnostic tool, which is usually complimented by isolating GAS from muscle biopsies [1, 3, 4]. Early and aggressive intravenous antibiotic therapy with penicillin and clindamycin is shown to be lifesaving if combined with early surgical debridement to decrease bacterial load [1]. Clindamycin has high soft tissue penetration and is effective against the large bacterial load [1, 5]. Moreover, clindamycin has been shown to inhibit toxin production and the subsequent release of superantigens involved in the pathogenesis of STSS [1, 10]. In addition to antibiotic therapy, aggressive surgical treatment should be instigated with early and appropriate debridement and, if required, limb amputation and intensive organ support [3].

With comparable pathology and management, outcome was vastly different between these two cases. It should be recognised that premorbid state and physical fitness influence outcome as suggested by our cases.

## 7. Learning Points from the Case

1. In SNM soft tissue changes are subtle and occur late compared with NF. A high index of suspicion for SMN in all cases presenting with acute disproportionate limb pain associated with systemic deterioration.
2. Early and aggressive antibiotic therapy with a combination of penicillin and clindamycin is imperative for suspected cases of SMN.
3. Early referral to a surgical team for aggressive excision and debridement of infected tissues is crucial and early amputation should be considered if indicated.

## Figures and Tables

**Table 1:** Blood results from patients 1 and 2 taken upon admission to our hospital before plastic surgery referral.

Test	Patient 1	Patient 2	Units	Range
Haemoglobin	15.3	12.0	g/dL	12–15
Sodium	127	125	mmol/L	135–145
Potassium	5.1	3.7	mmol/L	3.5–5.0
Urea	10.1	15.1	mmol/L	2.5–6.7
eGFR	34	19	mL/min/1.73 m^2^	
Creatinine	150	222	umol/L	54–145
Bilirubin	62	7	umol/L	3–17
ALT	249	52	IU/L	10–45
AST	817	—	IU/L	15–42
ALP	450	146	IU/L	75–250
Albumin	31	26	g/L	35–50
Creatine kinase	35040	1948	IU/L	24–195
Glucose	5.2	6.5	mmol/L	
INR	1.8	1.5	ratio	0.7–1.2
WCC	6.93	29.8	×10^9^/L	4.0–11.0
CRP	>156	>156	mg/L	0–8

eGFR: estimated glomerular filtration rate (automated); ALT: alanine aminotransferase; AST: aspartate aminotransferase; ALP: alkaline phosphatase; INR: international normalised ratio; WCC: white cells count; CRP: C-reactive protein.

**Table 2:** LRINEC scoring for patient 1 and patient 2. The table is modified from Wong et al., 2004 [13].

Parameter	Range	Score	Patient 1	Patient 2
CRP (mg/L)	<150	0		
	>150	4	4	4

WBC (cells/mm^3^)	<15	0	0	
	15–25	1		
	>25	2		2

HB (g d/L)	>13.5	0	0	
	11–13.5	1		1
	<13.5	2		

Creatinine	<141	0		
	>141	2	2	2

Sodium (mmol/L)	>135	0		
	<135	2	2	2

Glucose (mmol/L)	<10	0	0	0
	>10	1		

Total			**8**	**11**

## Appendix

For details see Figures 1, 2, and 3 and Tables 1 and 2.
